# Supplementary material for: Identification of Factors Linked to Higher Water-Deficit Stress Tolerance in Amaranthus hypochondriacus Compared to Other Grain Amaranths and A. hybridus, Their Shared Ancestor
Source: Plants (Basel). 2019 Jul 22;8(7):239. doi: 10.3390/plants8070239 (PMC6681232; doi:10.3390/plants8070239)
Supplement: Supplementary file 1 [file plants-08-00239-s001.pdf]

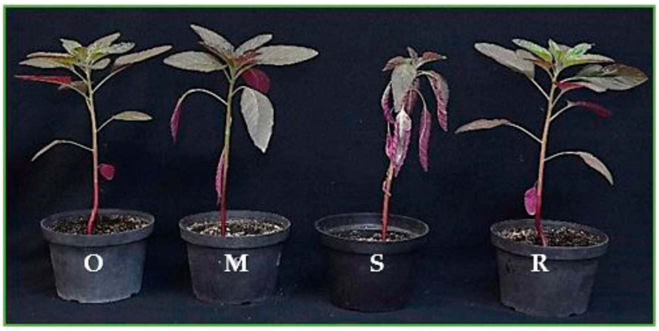

(A)

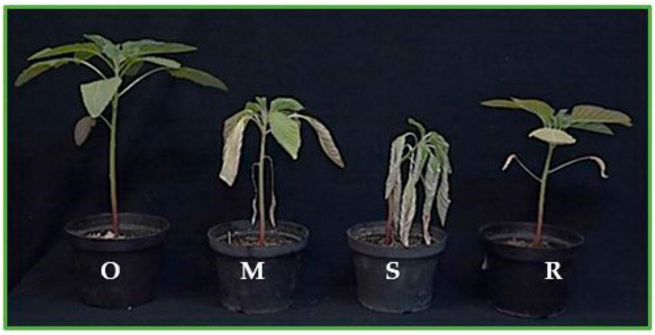

(B)

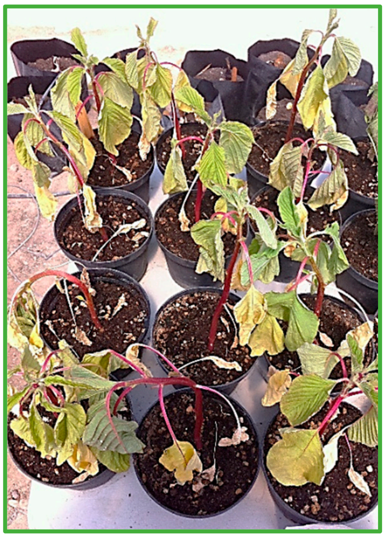

(C)

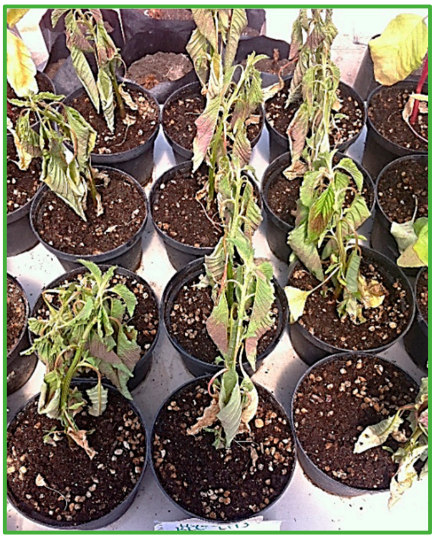

(D)

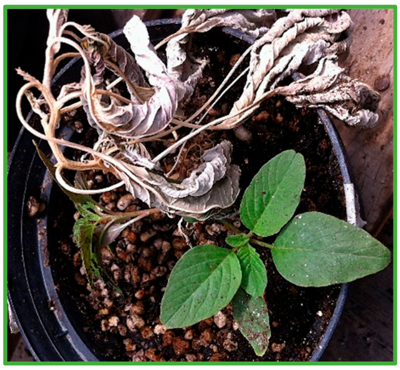

(E)

Figure S1. Response to water-deficit stress (WDS) in different amaranth species. Images of representative (A) *Amaranthus hypochondriacus* cv. Gabriela and (B) *A. cruentus* cv. Amaranteca plants used in the first selection experiment under optimal conditions [O], and 5 [M] and 10 [S] days after watering was withheld, and in [R], one day after watering was restored, after S. Panels (C) and (D) show *A. caudatus* and *A. hybridus* plants during one of the two formal WDS experiments performed under moderate WDS. Wilting is evident in both groups of plants even before the final 30% field capacity was reached. In panel (E), an image of the extreme desiccation suffered by *A. hybridus* plants during the preliminary 5- and 10-day WDS experiments. Also shown, is this species′ ability to partially recover several days after watering was restored.

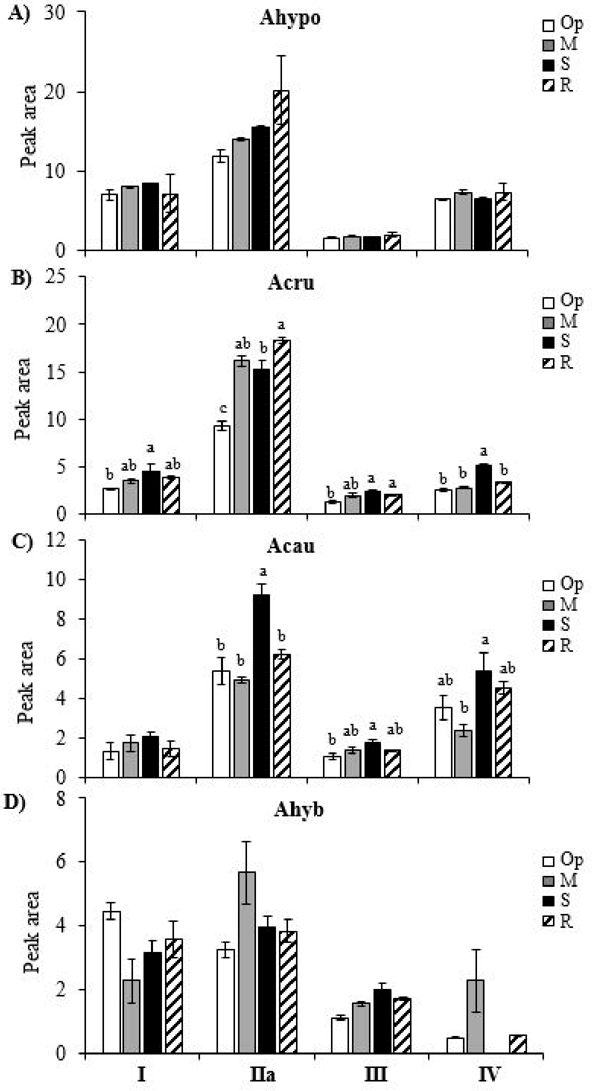

Figure S2. Unidentified RFO-like compounds quantified by HPAEC-PAD in leaf extracts of four species of amaranth plants. (**A**) *Amaranthus hypochondriacus* [Ahypo], (**B**) *A. cruentus* [Acru], (**C**) *A. caudatus* [Acau], and (**D**) *A. hybridus* [Ahyb]) growing in optimal conditions (Op; empty bars), subjected to moderate (M) or severe (S) water deficit stress (gray and black bars respectively) or allowed to recover from S, 1 day after normal watering was restored (R; striped bars). The unidentified compounds analyzed, possibly RFO with increased degree of polymerization, are labelled in Roman numerals in ascending order in correspondence with their longer elution times, as follows: peak **I** (16 min); peak **IIa** (21.2 min); peak **III** (31.4 min) and Peak **IV** (33.8 min). The bars represent peak areas. Different letters over the bars represent statistically significant differences at *P* ≤ 0.05 (Tukey Kramer test). Bars and error bars indicate mean values and ES, respectively (n = 3 pools of four plants each). The results shown are those obtained from a representative experiment that was repeated in the spring-summer and summer-autumn seasons. seasons of 2014, respectively, with similar results.

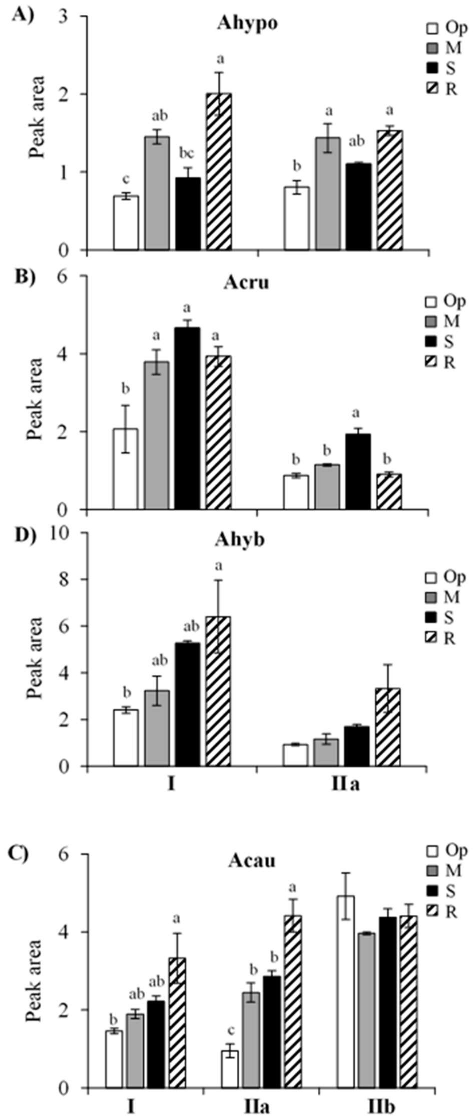

Figure S3. Unidentified RFO-like compounds quantified by HPAEC-PAD in root extracts of four species of amaranth plants. (**A**) *Amaranthus hypochondriacus* [Ahypo], (**B**) *A. cruentus* [Acru], (**C**) *A. caudatus* [Acau], and (**D**) *A. hybridus* [Ahyb]) growing in optimal conditions (Op; empty bars), subjected to moderate (M) or severe (S) water deficit stress (gray and black bars respectively) or allowed to recover from S, 1 day after normal watering was restored (R; striped bars). The unidentified compounds analyzed, possibly RFO with increased degree of polymerization, are labelled in Roman numerals in ascending order, in correspondence with their longer elution times, as follows: peak **I** (16 min); peak **IIa** (21.2 min), and Peak **IIb** (22.8 min). The bars represent peak areas. Different letters over the bars represent statistically significant differences at $P \leq 0.05$ (Tukey Kramer test). Bars and error bars indicate mean values and ES, respectively (n = 3 pools of four plants each). The results shown are those obtained from a representative experiment that was repeated in the spring-summer and summer-autumn seasons.

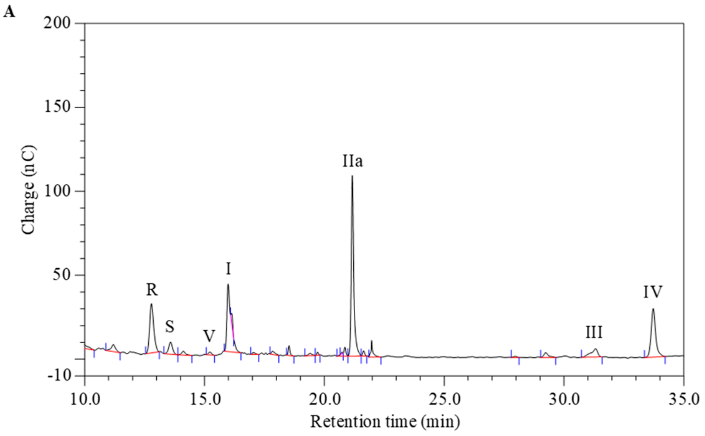

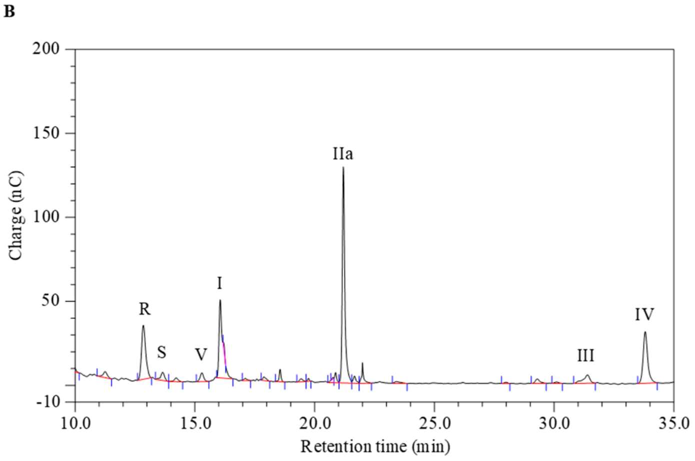

Figure S4. Representative HPAEC-PAD separation of soluble raffinose family oligosaccharides (RFOs) and unknown RFO-like compounds extracted from leaf extracts of *Amaranthus hypochondriacus*. The traces were obtained from plants growing in (**A**) optimal conditions or (**B**) subjected to severe water deficit stress. The peaks shown correspond to known raffinose (R), staquiose (S) and verbascose (V) oligosaccharides. Unknown RFO-like compounds were labeled with Roman numerals assigned according to their increasing retention time: I (16 min), IIa (21.2 min), III (31.4 min), and IV (33.8 min).

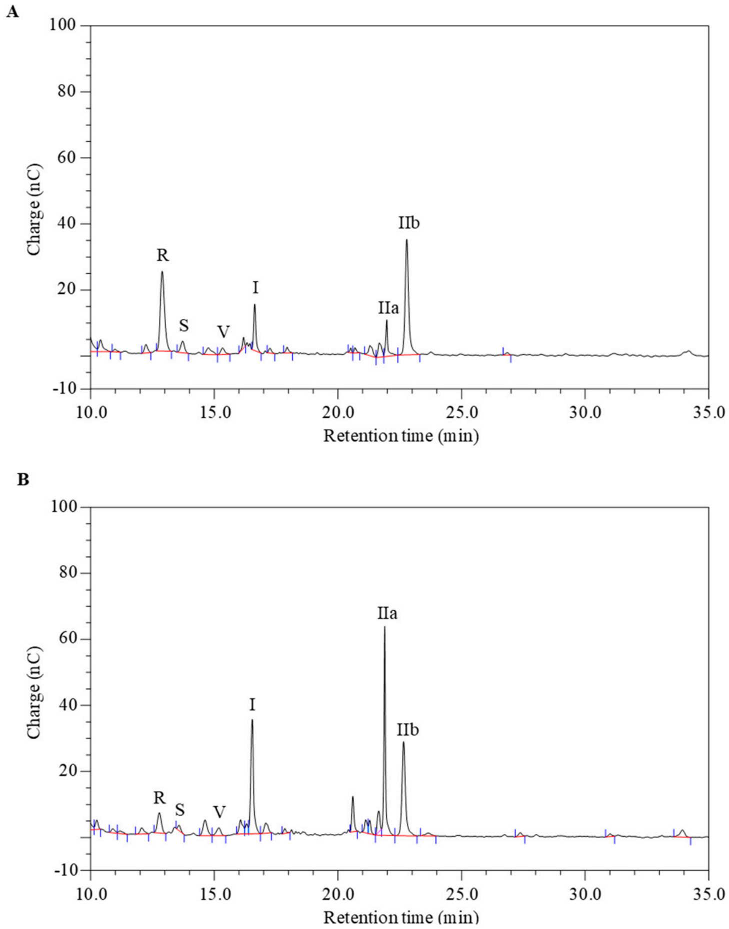

Figure S5. Representative HPAEC-PAD separation of soluble raffinose family oligosaccharides (RFOs) and unknown RFO-like compounds extracted from root extracts of *Amaranthus hypochondriacus*. The traces were obtained from plants growing in (**A**) optimal conditions or (**B**) subjected to severe water deficit stress. The peaks shown correspond to known raffinose (R), stachiose (S) and verbascose (V) oligosaccharides. Unknown RFO-like compounds were labeled with Roman numerals assigned according to their retention time: I (16 min), IIa (21.2 min), IIb (22.8 min).

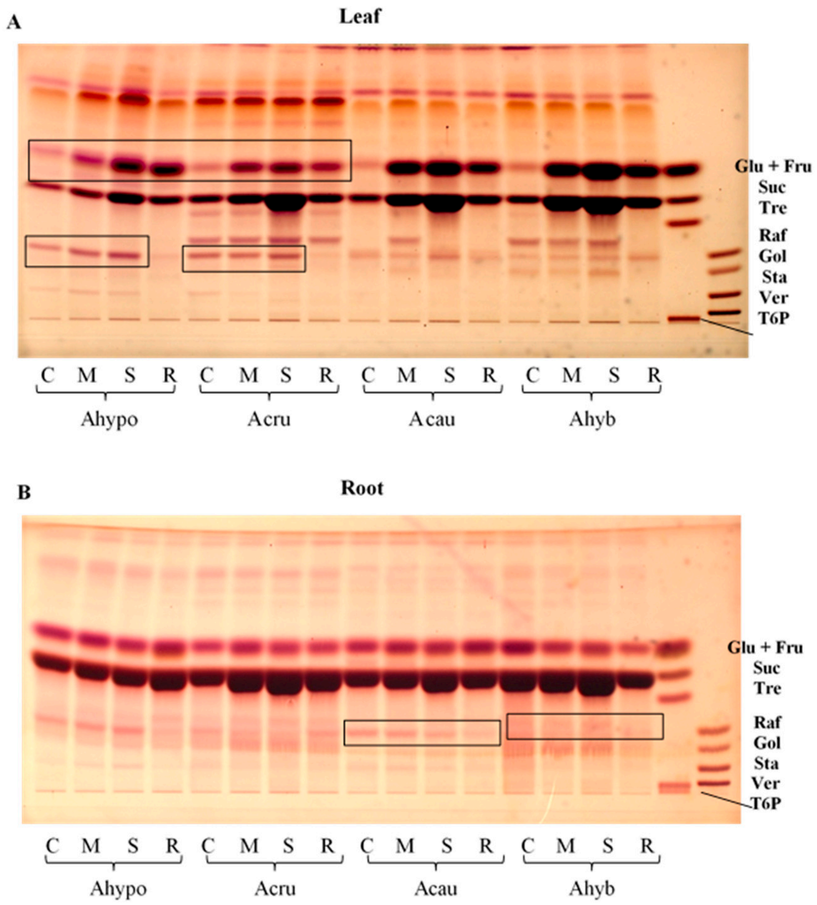

Figure S6. Thin layer chromatography separation of soluble non-structural carbohydrates and raffinose family oligosaccharides (RFOs). These were extracted from **(A)** leaves and **(B)** roots of four species of amaranth plants: *Amaranthus hypochondriacus* [Ahypo], *A. cruentus* [Acru], *A. caudatus* [Acau], and *A. hybridus* [Ahyb]) growing in optimal conditions (Op), subjected to moderate (M) or severe (S) water deficit stress or allowed to recover from S, 1 day after normal watering was restored (R). The position of the RFO and their respective precursor standards are shown in last two lanes on the right edge of the plate: These were the following: galactinol (Gol), raffinose (Raf), staquiose (Sta) and verbascose (Ver). Also included were glucose + fructose (Glu + Fru), sucrose (Suc), trehalose (Tre) and trehalose-6-phosphate (TGP) standards. Unidentified compounds, perhaps representing unknown RFOs that were constitutively accumulated in these tissues and/or were further increased in treated plants are enclosed in boxes.

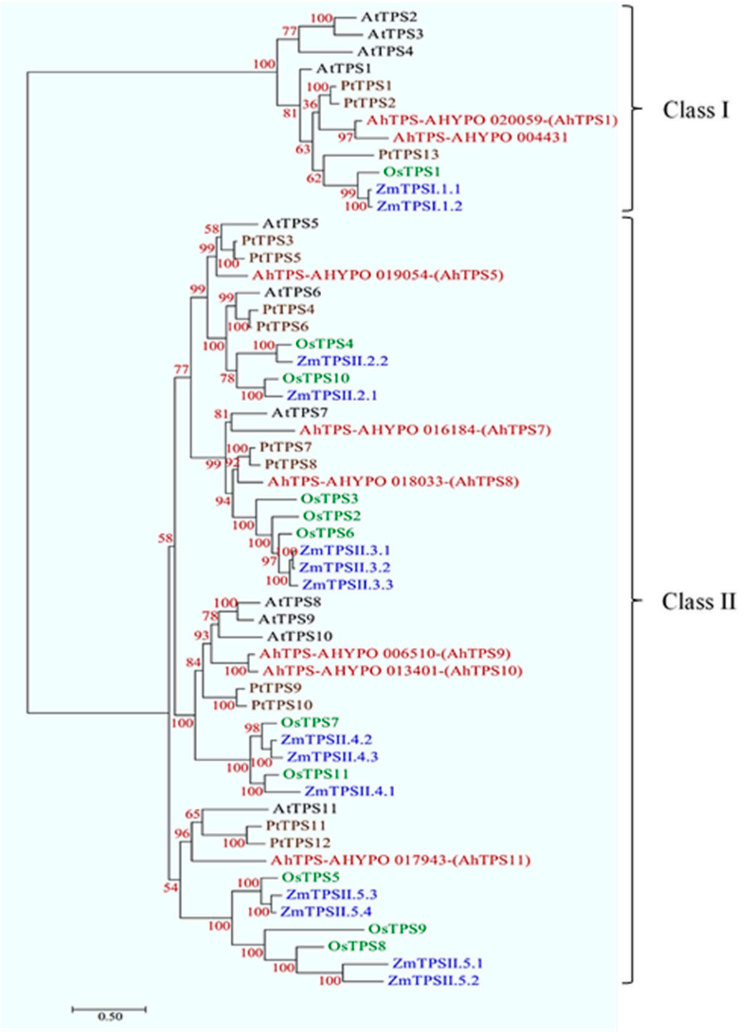

Figure S7. Phylogenetic analysis of the class I and II trehalose phosphate synthase proteins. The AhTPS proteins, shown in red text, were obtained from their respective genes, themselves contained in the *Amaranthus hypochondriacus* genome (Clouse et al., 2016). The phylogeny of AhTPS proteins with members reported in other plant species is shown. Abbreviations used are the following: Ah, *Amaranthus hypochondriacus*, At, *Arabidopsis thaliana* (black text); Os, *Oryza sativa* (green text); Pt, *Populus trichocarpa* (brown text), and Zm, *Zea mays* (blue text). The tree was obtained using the maximum likelihood method.

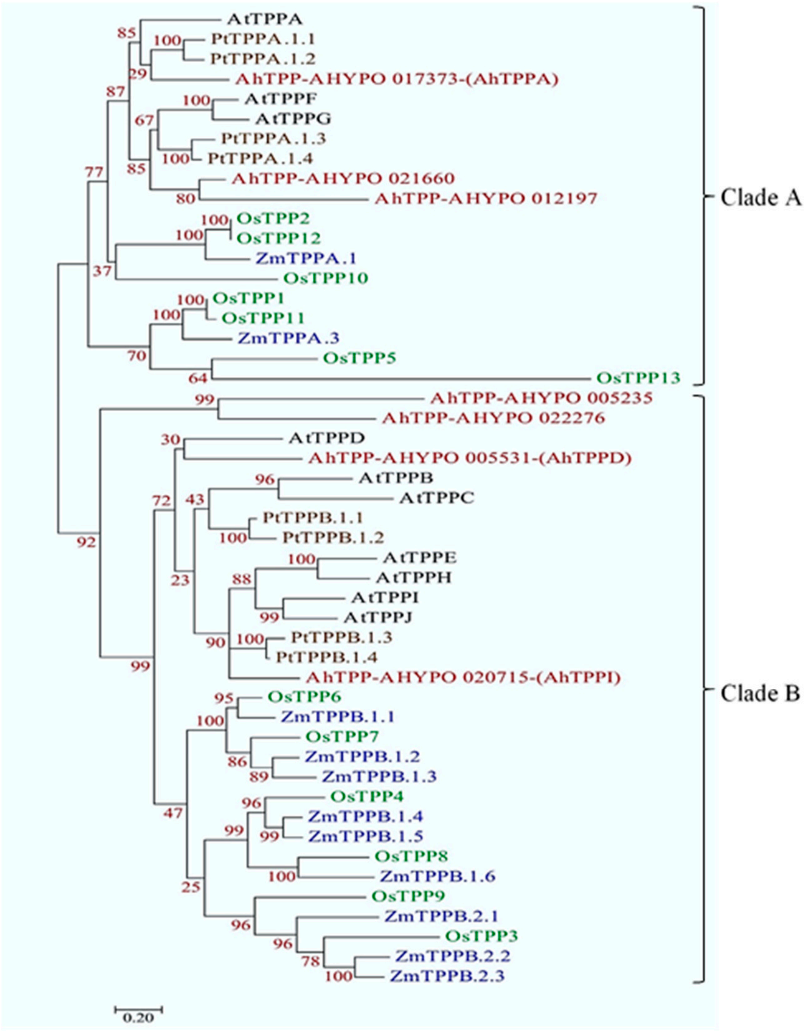

Figure S8. Phylogenetic analysis of the amaranth trehalose phosphate phosphatase proteins. The AhTPP proteins, shown in red text, were obtained from their respective genes, themselves contained in the *Amaranthus hypochondriacus* genome as reported by Clouse et al. [31]. The phylogeny of AhTPP proteins with members reported in other plant species is shown. Abbreviations used are the following: Ah, *Amaranthus hypochondriacus*, At, *Arabidopsis thaliana* (black text); Os, *Oryza sativa* (green text); Pt, *Populus trichocarpa* (brown text), and Zm, *Zea mays* (blue text). The tree was obtained using the maximum likelihood method.

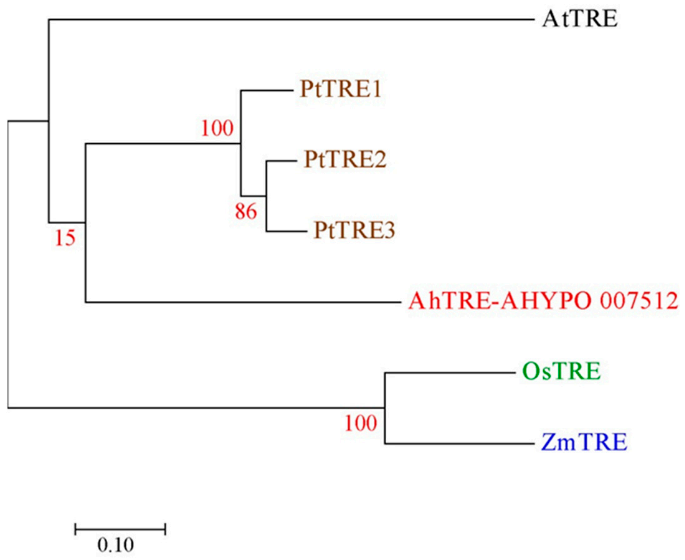

Figure S9: Phylogenetic analysis of the amaranth trehalase protein. The predicted AhTRE protein, shown in red text, was obtained from its respective gene, itself contained in the *Amaranthus hypochondriacus* genome as reported by Clouse et al. [31]. The phylogeny of the AhTRE protein with members reported in other plant species is shown. Abbreviations used are the following: Ah, *Amaranthus hypochondriacus*, At, *Arabidopsis thaliana* (black text); Os, *Oryza sativa* (green text); Pt, *Populus trichocarpa* (brown text), and Zm, *Zea mays* (blue text). The tree was obtained using the maximum likelihood method.

Table S1. Relative expression levels[1] of selected *SnRK, SnGRIK* and *SnRK2* genes in amaranth leaves. Gene expression was determined in leaves of four *Amaranthus* species subjected to two levels of water-deficit stress (moderate [M] and severe [S]) and to subsequent recovery ([R]). Induced (normalized expression values ≥ 2.0; in normal text) and repressed (normalized expression values ≤ 0.5; in italicized text) expression values are emphasized in bold.

Gene	*A. hypochondriacus*			*A. cruentus*			*A. caudatus*			*A. hybridus*		
	M	S	R	M	S	R	M	S	R	M	S	R
AhGRIK2-like	1.281	1.163	1.483	0.777	0.846	0.532	1.048	**1.803**	**1.628**	0.966	**2.744**	1.443
AhSnRK1	0.783	**0.495**	0.648	0.613	0.707	0.741	1.286	1.323	1.276	1.437	1.299	0.885
AhSnRK2.1	**0.447**	**0.322**	0.586	**0.467**	**0.364**	0.547	0.805	0.702	1.216	0.54	0.504	0.853
AhSnRK2.2	1.301	0.922	1.053	0.806	**0.485**	0.595	1.224	1.334	1.198	**2.239**	**1.789**	0.964

Calculated according to the comparative cycle threshold method (Livak and Schmittgen, 2001) using the *AhACT7*, *AhEF1a* and *AhβTub5* amaranth genes for data normalization.

Table S2. Relative expression values[1] of selected *SnRK, SnGRIK* and *SnRK2* genes in amaranth roots. Gene expression was determined in roots of four *Amaranthus* species subjected to two levels of water-deficit stress (moderate [M] and severe [S]) and to subsequent recovery ([R]). Induced (normalized expression values ≥ 2.0; in normal text) and repressed (normalized expression values ≤ 0.5; in italicized text) expression values are emphasized in bold.

Gene	*A. hypochondriacus*			*A. cruentus*			*A. caudatus*			*A. hybridus*		
	M	S	R	M	S	R	M	S	R	M	S	R
AhGRIK2-like	**4.857**	**3.352**	**3.100**	0.956	**1.516**	0.842	0.617	1.031	0.881	0.827	1.156	0.652
AhSnRK1	1.183	**1.933**	1.464	0.544	1.408	0.791	**0.297**	0.712	**0.162**	**1.818**	1.371	0.801
AhSnRK2.1	0.672	0.688	0.638	0.964	0.918	0.638	0.952	0.957	0.929	0.604	0.679	0.392
AhSnRK2.2	0.925	0.654	0.981	0.962	1.338	1.220	0.952	0.957	0.929	1.103	1.074	1.172

Calculated according to the comparative cycle threshold method (Livak and Schmittgen, 2001) using the *AhACT7*, *AhEF1a* and *AhβTub5* amaranth genes for data normalization.

Table S3. List of qPCR primers used in this study.

Gene		Sequence
AhTPS1	Forward	CTCCTTACAGTTGAGAAATCGC
	Reverse	TAACACTCTTGACCCAGATCG
AhTPS5	Forward	AACATCCTTAGTATCTCGCCTG
	Reverse	AGAACAACTCCTTCACTCTCG
AhTPS7	Forward	AGGAAGCGATATTGAGGAGA
	Reverse	GCAATGGTGAAGTAAGCGAT
AhTPS8	Forward	CACCATTAGTAGGTCCAACAG
	Reverse	CAAGTGATGATGAGCCAGG
AhTPS9	Forward	TTCTTGCTCTGCCACTCAC
	Reverse	ATTGTCTCGGCGTATAGGA
AhTPS10	Forward	TCCTTGAAAGACGGTCAGAAT
	Reverse	CTGCTTGTGTGCTTGAAGTTA
AhTPS11	Forward	AACAACGACAGGCTCATTAG
	Reverse	GGCATTATCAAGACTCCGA
AhTPPA	Forward	TGTTCGTATTACTGCTTGGC
	Reverse	TGAGAGGGCTGACGGATA
AhTPPD	Forward	GCAGATGGATGATTGACC
	Reverse	TTCCAACTCCTCCTCCTTC
AhTPPI	Forward	TTGGATTACGATGGCACTCT
	Reverse	GGCACCTTCCACTCACTATT
AhTRE	Forward	AACAACGGTAACGGTAGGAG
	Reverse	GCAAGCACAGATCAATGC
AhGOLS1	Forward	AGCCCAACTACACCCTTTATG
	Reverse	TAATGGCACCAACAAACTTCG
AhGOLS2	Forward	TCCTCCTACTCAAACTATCCAA
	Reverse	AAAGGATCTAAGCAGCAGAAG
AhReFS	Forward	AAGCAGTTGAGTCACTATTCCA
	Reverse	AGATGGTTCGTCTTGGCATAA
AhSTaS	Forward	CAATCCAAGGCAAATCAAACAC
	Reverse	TCAAATGAGGCACCAAAGAAG
AhGRIK2-like	Forward	CTCCTGTAGTGTCTCTCCGATA
	Reverse	CCCGAATGTTGCTTAGGT
AhSnRK1	Forward	GGACCTCAGATGCTCTTCCTT
	Reverse	TGAAACACGACCATACATCAT
AhSnRK2.1	Forward	ATCCCAAAGTCTGAACAAGTA
	Reverse	CGGCTCATTACAATCGTTT
AhSnRK2.2	Forward	TCACAACAGAGGGACAATAAA
	Reverse	GCTCTCTTCTTGGGTGGA
AhRAB18	Forward	GCCCAGTGAAACAAGCAATAC
	Reverse	GCCAGAGACTCCGTAAACTTC
AhDREB2C	Forward	TAACTCCGCAACAACAACACC
	Reverse	TCGATTCGACCAACTGACTGA
AhABI5	Forward	TTTCTGAACTTCTTGCTCCAACT
	Reverse	AGAGGAGACAAAGGCGTATGA
AhLEA14	Forward	CCCGACAATATCTCCCTGGTA
	Reverse	ACGTACTCTGCTAAAGTTGGTG
AhActin (ACT)	Forward	CGTGACCTGACTGATTACCTTA
	Reverse	GCTCGTAGTTCTTCTCAATGGC
Ahβ-Tubulin (TUB)	Forward	TCTCAGCAGTATGTCTCCCTCA
	Reverse	TCTACTTCTTTGGTGCTCATCTT
AhElongation factor 1α (*EF1α*)	Forward	GCCAAATATCTAAGAAACAAATGC
	Reverse	TAGCACAACCACATGATATTTCTT

Table S4. Genes found to be differentially expressed in *A. hypochondriacus* plants subjected to water-deficit stress. Induced (relative expression value ≥ 1.5) or repressed (relative expression value ≤ 0.5) genes in each category are shown.

	**CELL WALL/ CYTOSKELETON RELATED**		
	**XYLOGLUCANS**		
**0.1**	gb|AAB39950.1| xylucan endotransglycosylase		
**0.2**	gb|AAK51119.1| xylucan endo-transglycosylase [*Carica papaya*]		
**0.2**	gb|ABB72441.1| xylucan endotransglucosylase [*Betula pendula*]		
**0.4**	Bifunctional endo-1,4-beta-xylanase XylA precursor		
**0.1**	gb|ABV27475.1| fasciclin-like arabinogalactan protein 4		
**0.3**	gb|AAM62616.1| arabinogalactan protein-like [*Arabidopsis thaliana*]		
**0.1**	gb|ABV27490.1| fasciclin-like arabinogalactan protein 19 [*Gossypum* spp.]		
	**PECTIN MATRIX**		
**0.1**	gb|AAK66161.1| pectate lyase [*Fragaria x ananassa*] 430 e-118	**3.5**	gb|ABG47806.1| pectin methlyesterase inhibitor protein 1 [*Capsicum* spp.]
**0.2**	dbj|BAE98780.1| pectinesterase like protein [*Arabidopsis thaliana*]	**2.3**	gb|ABS18954.1| PGIP2 [*Populus deltoides*]
**0.1**	ref|NP_200149.1| ATPMEPCRF; pectinesterase [*Arabidopsis thaliana*]		
**0.5**	gb|AAA53547.1| polygalacturonase inhibitor protein 231		
**0.2**	ref|NP_566170.1| GAUT9 (Galacturonosyltransferase 9); polygalacturonase		
**0.2**	ref|NP_196051.2| pectate lyase family protein [*Arabidopsis thaliana*]		
**0.3**	ref|NP_001118545.1| GAUT13 (Galacturonosyltransferase 13) [*Arabidopsis thaliana*]		
**0.4**	ref|NP_186753.2| GAUT13 (Galacturonosyltransferase 13); polygalacturonase		
**0.3**	ref|NP_194113.1| glycoside hydrolase family 28 protein / polygalacturonase		
**0.5**	gb|ACF05806.1| Pectin acetyl esterase [*Litchi chinensis*]		
**0.2**	gb|AAT40110.1| putative UDP-glucuronate decarboxylase 4 [*Nicotiana* spp.]		
**0.1**	dbj|BAD91079.1| beta-D-galactosidase [*Pyrus pyrifolia*]		
**0.3**	ref|NP_197193.1| ATCSLD2 (Cellulose synthase-like D2); cellulose.	**6.5**	gb|AAZ79231.1| cellulose synthase-like protein CslG [*Nicotiana tabacum*]
**0.3**	sp|A2YU42.1|CSLD2_ORYSI RecName: Full=Cellulose synthase-like protein	**1.9**	gb|AAK49454.1|AF304374_1 cellulose synthase catalytic subunit
**0.5**	gb|AAY60847.1| cellulose synthase 5 [*Eucalyptus grandis*]		
**0.1**	ref|NP_193893.1| glycine-rich protein [*Arabidopsis thaliana*]	**2.2**	gb|ABG76000.1| GRP-like protein 2 [*Gossypium hirsutum*]; glycine-rich protein

**0.2**	ref|NP_190109.1| hydroxyproline-rich glycoprotein family protein		
**0.3**	gb|AAM65462.1| glycine rich protein-like [*Arabidopsis thalian*a]		
**0.2**	dbj|BAC67189.1| expansin [*Pyrus communis*]	**2.8**	ggb|AAR09170.1| alpha-expansin 3 [*Populus tremula x Populus tremula*]
**0.3**	gb|AAN87551.1|AF376053_1 gamma-tubulin [*Lupinus albus*]	**2.1**	ref|NP_567094.1| ADL4/ADLP2/DRP1E/EDR3 (DYNAMIN-LIKE PROTEIN 4)
**0.3**	ref|NP_176252.1| dynamin family protein [*Arabidopsis thaliana*]	**1.9**	gb|AAF35902.1|AF230333_1 expansin 3 [*Zinnia elegans*]
**0.1**	gb|ABO28522.1| kinesin-related protein [*Gossypium hirsutum*]	**5.2**	sp|Q9ZPN9.1|TBB2_ELEIN Tubulin beta-2 chain (Beta-2-tubulin)
**0.2**	dbj|BAB40710.1| BY-2 kinesin-like protein 10 [*Nicotiana tabacum*]		
**0.4**	gb|ACG39191.1| actin-1 [*Zea mays*]		
**0.5**	dbj|BAA82639.1| Beta-tubulin [*Zinnia elegans*]		
**0.1**	sp|P51074.2|ANX4_FRAAN RecName: Full=Annexin-like protein		
**0.1**	sp|P83304.1|LEC_PARPC RecName: Full=Mannose/glucose-specific lectin	**4.1**	dbj|BAB18761.1| lectin [*Helianthus tuberosus*]
**0.1**	emb|CAA61158.1| SIEP1L protein [*Beta vulgaris* subsp. *vulgaris*], LECTIN	**3.2**	gb|ABA94728.1| Jacalin-like lectin domain containing protein
**0.3**	gb|AAT37529.1| purple acid phosphatase 1 [*Solanum tuberosum*]		
**0.4**	gb|ABD28734.1| UDP-N-acetylglucosamine transferase subunit ALG14		
**0.2**	gb|AAL77689.1| AT5g55180/MCO15_13 [*Arabidopsis thaliana*]; O-glycosyl transferase family 17		
**0.4**	gb|ACG27548.1| heparanase-like protein 2 precursor [*Zea mays*]		
**0.3**	gb|AAP97493.1| UDP-Glc-4-epimerase [*Solanum tuberosum*]		
		**11.9**	ref|NP_176737.2| ATHEX1/HEXO3 (BETA-HEXOSAMINIDASE 3);
		**4.5**	ref|NP_683538.1| glycosyl hydrolase family 17 protein [*Arabidopsis thaliana*]
		**2.1**	gb|ACC91241.1| aldose 1-epimerase family protein [*Arabidopsis thaliana*]
**PRIMARY/ SECONDARY METABOLISM**			
**0.5**	gb|AAB99757.1| malate dehydrogenase precursor [*Medicago sativa*]; **partitioning of carbon and energy in leaves**	**4.5**	sp|P46488.1|MDHG_CUCSA RecName: Full=**Malate dehydrogenase**, glyoxysomal
		**5.9**	sp|Q05145.1|MDHP_MESCR RecName: Full=**Malate dehydrogenase** [NADP]
		**8.6**	emb|CAD33240.1| putative mitochondrial **NAD-dependent malate dehydrogenase**
**0.1**	ref|NP_190421.1| CYP94B3 (cytochrome P450, family 94, subfamily	**1.6**	ref|NP_563789.1| SMO2-2 (sterol 4-alpha-methyl-oxidase 2); C-4 m.
**0.1**	ref|ZP_01308190.1| methyl-accepting chemotaxis domain/unknown domain	**7.1**	sp|P46255.1|DCAM_SPIOL RecName: Full=S-adenosylmethionine decarboxylase

**0.5**	sp|P49297.1|ACEA_SOLLC RecName: Full=Isocitrate lyase (Glioxylate cycle associate with senescence)	**1.5**	pdb|2AKJ|A Chain A, Structure of spinach Nitrite Reductase; **coordination of nitrogen and carbon metabolism**
**0.1**	gb|AAP74755.1| chalcone synthase [*Gypsophila paniculata*]	**2.8**	ATPDX2/EMB2407/PDX2 (PYRIDOXINE BIOSYNTHESIS 2)
**0.3**	ref|NP_197195.2| AAA-type ATPase family protein [*Arabidopsis thaliana*]	**3.1**	gb|ACF17659.1| putative 3-isopropylmalate dehydrogenase small subunit
**0.1**	ref|NP_173857.2| methyltransferase [Arabidopsis thaliana]	**3.9**	dbj|BAD80839.1| 2-Hydroxyisoflavanone dehydratase [*Glycyrrhiza* spp.]
**0.1**	dbj|BAE92730.1| 3-hydroxy-3-methylglutaryl coenzyme A reductase	**1.5**	emb|CAC33003.1| urease accessory protein G [*Solanum tuberosum*]; **nitrogen recycling**
**0.1**	gb|AAP96920.1| pyruvate decarboxylase [*Dianthus caryophyllus*]	**1.6**	gb|ABC02881.1| ACS2 [Ricinus communis]; Acyl-CoA synthetase
**0.3**	gb|ABK76265.1| beta-amyrin synthase [Vaccaria hispanica], saponins, **drought stress**	**2.1**	gb|AAT12274.1| obtusifoliol 14alpha-demethylase [*Solanum chacoense*]
**0.2**	gb|AAC06319.1| putative cinnamyl alcohol dehydrogenase [*Malus* spp.]	**5.3**	ref|NP_172724.2| membrane bound O-acyl transferase (MBOAT) family
**0.3**	ref|NP_188226.1| A37 (PYRIDOXINE BIOSYNTHESIS 1.2)	**2.6**	ref|NP_567228.1| epoxide hydrolase, putative [*Arabidopsis thaliana*]
**0.2**	sp|Q07510.1|FABH_SPIOL RecName: Full=3-oxoacyl-[acyl-carrier-protein	**3.1**	sp|Q84KI6.1|SQD1_SPIOL RecName: Full=UDP-sulfoquinovose synthase.
**0.3**	gb|ABC74567.1| acetoacetyl-CoA thiolase [*Picrorhiza kurrooa*]	**5.5**	emb|CAC91565.1| hydroperoxide lyase [*Nicotiana attenuata*]
**0.3**	sp|O65162.1|CAMT_MESCR RecName: Full=Caffeoyl-CoA O-methyltransferase	**1.9**	gb|AAR29980.1| sterol delta-7 reductase [*Tropaeolum majus*]
**0.3**	emb|CAD23248.1| squalene monooxygenase 2 [*Medicago truncatula*]	**1.9**	ref|NP_566011.1| MTHFR2 (METHYLENETETRAHYDROFOLATE REDUCTASE 2)
**0.3**	gb|ACC63870.1| coumaroyl 3-hydroxylase [*Populus trichocarpa*]	**4.5**	gb|ACB56924.1| glycosyltransferase UGT88A8 [*Hieracium pilosela*]
**0.4**	dbj|BAF98285.1| diphosphomevelonate decarboxylase [*Hevea brasiliensis*]		
**0.5**	gb|AAC49114.1| acetyl-CoA carboxylase biotin-containing subunit		
**0.2**	gb|AAP96921.1| alcohol dehydrogenase [*Dianthus caryophyllus*]		
**0.5**	dbj|BAF98279.1| hydroxymethylglutaryl-CoA synthase [*Hevea brasiliensis*]		
**0.1**	tpe|CAI56335.1| TPA: isoflavone reductase-like protein 6 [*Vitis* spp.]		
**0.2**	gb|AAF76143.1|AF258339_ 4-diphosphocytidyl-2-C-methyl-D-erythritol kinase, chloroplastic/chromoplastic		
**0.1**	gb|ABB89006.1| CXE carboxylesterase [*Malus pumila*]		
	**RNA-DNA BINDING PROTEINS**		
**0.1**	ref|NP_565175.1| RNA recognition motif (RRM)-containing protein	**1.5**	chloroplast RNA binding protein [*Arabidopsis thaliana*].
**0.1**	ref|NP_177158.1| DNA-binding family protein [*Arabidopsis thaliana*]	**3.1**	dbj|BAG09558.1| chloroplast RNA binding protein [*Mesembryanthemum* sp.]
**0.5**	dbj|BAA22083.1| RNA binding protein [*Nicotiana sylvestris*]		
	**PHYTOHORMONE-RELATED (development/ stress)**		
**0.3**	ref|NP_200336.1| IAMT1 (IAA CARBOXYLMETHYLTRANSFERASE 1); converts IAA to methyl-IAA ester (MeIAA) *in vitro*,	**5.0**	gb|ACL81167.1| **auxin-responsive** protein IAA1 [*Mirabilis jalapa*]

	suggesting that methylation of IAA plays an important role in regulating plant development and **auxin** homeostasis.		
**0.2**	gb|ABL67651.1| putative **auxin-repressed**/dormancy-associated protein		
**0.4**	gb|ACL81159.1| putative **auxin/aluminum**-responsive protein		
**0-4**	gb|ABN81351.1| **auxin influx transport** protein [*Casuarina glauca*]		
**0.2**	gb|ABB52080.1| putative 9-cis epoxycarotenoid dioxygenase (**ABA biosynthesis**)	**2.6**	sp|Q40412.1|ABA2_NICPL RecName: Full=Zeaxanthin epoxidase; **ABA biosynthesis**
		**2.1**	gb|AAP41850.1| 1-aminocyclopropane-1-carboxylate oxidase [*Hevea* sp.] (**ethylene biosynthesis**)
**0.1**	gb|AAP83138.1| lipoxygenase [*Nicotiana attenuata*]	**6.5**	gb|AAP83137.1| lipoxygenase [*Nicotiana attenuata*] (**jasmonic acid**)
**0.3**	ref|NP_568914.1| **gibberellin-regulated** family protein [*Arabidopsis thaliana*]		
**0.3**	emb|CAB45241.1| GEG protein [Gerbera hybrid cultivar]; Gerbera hybrid homolog of the **gibberellin [GA]–stimulated transcript 1** [GAST1] from tomato		
**0.1**	ref|NP_191705.1| BRH1 (**BRASSINOSTEROID-RESPONSIVE RING-H2**); protein		
**0.1**	ref|NP_197535.2| TIP1 (TIP GROWTH DEFECTIVE 1) [*Arabidopsis thaliana*]	**5.6**	gb|ACC95130.1| COL2 [*Beta vulgaris* subsp. vulgaris]; **Constans-like protein**
		**6.7**	ref|NP_569012.2| **GRF8 (GENERAL REGULATORY FACTOR 8); 14:3:3. This protein is reported to interact with the BZR1 transcription factor involved in brassinosteroid signaling and may affect the nucleocytoplasmic shuttling of BZR1.**
		**7.6**	ref|NP_851274.1| **GRF8 (GENERAL REGULATORY FACTOR 8).**
	**STRESS-RELATED**		
**0.1**	sp|P09887.1|HS22C_SOYBN RecName: Full=**Small heat shock protein**	**2.4**	gb|AAB62947.1| LTCOR11 [*Lavatera thuringiaca*], **cold-responsive**
**0.1**	ref|NP_565824.1| **DNAJ heat shock N-terminal domain-containing protein**	**2.2**	dbj|BAB70509.1| DNAJ homoue [Oryza sativa]
**0.3**	ref|NP_195570.1| ATFP6 (FARNESYLATED PROTEIN 6); metal ion binding; **ACBP2-overexpressing plants were more tolerant to hydrogen peroxide** than wild-type plants, further supporting a role for ACBP2 in **post-stress membrane repair.**		ref|NP_568342.1| ENH1 (ENHANCER OF SOS3-1); metal ion binding, **enhanced salinity tolerance by excluding Na+ from the cytosol**
**0.4**	gb|ACK99529.1| **LEA protein** [*Knorringia sibirica*]	**3.7**	gb|AAT01418.1| putative stress-responsive protein [*Tamarix* sp.]
**0.1**	gb|ABB02409.1| chloroplast lipocalin [*Ipomoea nil*]		gb|AAO72532.1| aldehyde dehydrogenase 1 precursor [*Lotus* sp.]; **Different plant ALDH genes have been reported to respond to environmental stress conditions such as dehydration, salinity, or excessive light**

**0.3**	gb|AAC69177.1| peroxisomal targeting signal 1 receptor	**1.7**	gb|ACF06568.1| disulfide-isomerase precursor-like protein; **chaperone**
**0.3**	ref|NP_174368.1| CTP synthase, putative / UTP--ammonia ligase, **glutamine synthetase, salt stress**	**1.6**	ref|NP_175812.1| ALDH7B4 (**ALDEHYDE DEHYDROGENASE** 7B4)
**0.4**	ref|NP_030521.1| **dehydration-responsive family protein** [*Arabidopsis thaliana*]	**1.6**	gb|AAX09646.1| **aldehyde dehydrogenase** family 7 member A1
**0.3**	ref|NP_001119417.1| glucose-methanol-choline (GMC) oxidoreductase	**2.1**	ref|NP_192626.1| arginase, putative [*Arabidopsis thaliana*]
**0.1**	emb|CAR67806.1| **Chaperone protein htpG** (Heat shock protein htpG)	**2.2**	emb|CAC85228.1| **salt tolerance protein 2** [*Beta vulgaris*]
**0.1**	ref|NP_179374.1| ERD7 (**EARLY-RESPONSIVE TO DEHYDRATION 7**) [*Arabidopsis thaliana*]	**3.9**	ref|NP_564093.1| **wound-responsive family protein** [*Arabidopsis thaliana*]
**0.1**	gb|ACH87168.1| **senescence-related protein** [*Camellia sinensis*]	**3.5**	gb|AAR17080.1| **heat shock protein 70-3** [*Nicotiana tabacum*]
**0.4**	gb|ABX60218.1| **heat shock protein** [*Ammopiptanthus mongolicus]*	**3.8**	sp|P17202.1|BADH_SPIOL RecName: Full=**Betaine aldehyde dehydrogenase**
**0.2**	dbj|BAA11766.1| high mobility group protein [*Canavalia gladiata]*	**4.9**	gb|ABB02383.1| **temperature-induced lipocalin'** [Solanum *lycopersicum*]
**0.1**	ref|NP_568974.1| ATFP3 (*Arabidopsis thaliana* farnesylated protein	**4.1**	gb|AAA87042.1| **putative 32.7 kDa jasmonate-induced protein** [*Hordeum vulgare*]
**0.5**	gb|AAA61609.1| pyrophosphatase [*Beta vulgaris*]	**10.8**	gb|ABC26011.1| **carotenoid cleavage dioxygenase 4a** [*Citrus clementine*]

**TRANSCRIPTION FACTORS**			
**0.1**	gb|ABO48369.1| TINY-like protein 2 [*Populus trichocarpa],* **AP2/ERF**		
**0.1**	ref|NP_188299.1| **ATEBP/ERF72/RAP2.3** (RELATED TO AP2 3); DNA binding		
**0.1**	gb|AAV66332.1| **ethylene response factor 1** [*Cucumis sativus*]		
**0.2**	gb|AAK92213.1| **bZIP transcription factor BZI-2** [*Nicotiana tabacum*]		
**0.5**	**ABSCISIC ACID-INSENSITIVE 5-like protein 5//AREB-like protein//bZIP transcription factor//abscisic acid responsive elements-binding factor 2**		

**0.1**	ref|NP_849712.1| transcription regulator [*Arabidopsis thaliana*]	**2.1**	gb|ABV53916.1| **GAGA-motif binding** transcriptional activator
**0.1**	gb|AAY40250.1| Cys2/His2 zinc-finger transcription factor [*Silene latifolia*]	**3.5**	gb|ABN05686.1| **Zinc finger, CCHC-type** [*Medicago truncatula*]
**0.2**	dbj|BAD06717.1| WRKY transcription factor 1 [*Spinacia oleracea*]		
**0.2**	gb|AAY90122.1| basic helix-loop-helix transcription factor protein		
**0.5**	gb|AAZ30376.1| PHB1 [*Nicotiana benthamiana*], prohibitin		

**TRANSPORT**			
**0.3**	dbj|BAD91179.1| putative mitochondrial dicarboxylate transporter	**2.6**	ref|NP_187290.1| integral membrane family protein [*Arabidopsis thaliana*]
**0.1**	gb|AAT35231.1| nodulin 26-like protein [*Medicago truncatula*]	**1.9**	sp|P93690.1|SECY_SPIOL RecName: Full=Preprotein translocase subunit
**0.1**	ref|NP_200682.1| nuclear movement family protein [*Arabidopsis thaliana*]	**2.5**	sp|P42767|PIP1_ATRCA **Aquaporin PIP-type**
**0.4**	dbj|BAB11940.1| **Na/H antiporter** Nhx1 [*Atriplex gmelini*]	**1.5**	ref|NP_566180.1| **integral membrane family protein** [*Arabidopsis thaliana*]

**0.4**	gb|AAD31848.1|AF133532_1 **water channel protein MipK** [*Mesembryanthemum crystallinum*]	**2.1**	ref|NP_201094.1| permease, putative [*Arabidopsis thaliana*]
**0.2**	sp|Q43748.1|NLTP_BETVU RecName: Full=Non-specific **lipid-transfer**	**5.1**	gb|AAB67869.1| **plasma membrane major intrinsic protein 2** [*Beta vulgaris*]
**0.3**	gb|AAB95302.1| putative **nitrate transporter** [Arabidopsis thaliana]	**2.1**	dbj|BAD29977.1| putative **ammonium transporter** [*Camellia sinensis*]
**0.1**	ref|NP_179257.1| nodulin family protein [*Arabidopsis thaliana*]	**1.5**	ref|NP_566806.1| YSL6 (YELLOW STRIPE LIKE 6); **oligopeptide transporter**
**0.3**	ref|NP_568091.2| **CAX4 (cation exchanger 4); cation:cation antiporter**	**4.6**	emb|CAC84547.1| **dicarboxylate/ tricarboxylate carrier** [*Nicotiana* spp.]
		**4.5**	emb|CAD92450.1| **amino acid permease 6** [*Brassica napus*]
		**2.1**	gb|ACF06527.1| **secretory carrier membrane protein** [*Elaeis* spp.]
		**5.5**	emb|CAN99590.1| **Na+/H+ antiporter** [*Mesembryanthemum crystallinum*]
		**6.7**	dbj|BAD91181.1| putative mitochondrial adenylate transporter.
		**7.1**	|BAD91180.1| putative plastidic ANT1 - **ADP,ATP carrier protein** – [*Mesembryanthemum crystallinum*]
		**15.4**	ref|NP_182239.1| ARF1A1c (**ADP-RIBOSYLATION FACTOR 1); GTP binding**]
	**CARBOHYDRATE METABOLISM**		
**0.2**	gb|ABR87939.1| **sucrose synthase 1** [*Beta vulgaris*]	**1.8**	ref|NP_195020.1| GAMMA-VPE (**Vacuolar processing enzyme gamma**)
**0.1**	ref|YP_534971.1| neopullulanase / cyclomaltodextrinase / maltoge.	**1.5**	dbj|BAD93291.1| **beta-amylase** [*Glycine max*]
**0.2**	emb|CAA59464.1| alpha-glucan phosphorylase [*Spinacia oleracea*]	**2.7**	gb|AAY89374.1| **beta-amylase 1** [*Nicotiana langsdorffii x Nicotiana*]
		**4.9**	dbj|BAA09462.1| **beta-amylase** [*Glycine max*]
		**6.9**	gb|ACH72975.1| **granule-bound starch synthase** [*Nelumbo nucifera*]
		**7.7**	dbj|BAD91175.1| **plastidic glucose 6-phosphate/phosphate translocator**
		**2.1**	gb|ACF04278.1| UTP-glucose 1 phosphate uridylyltransferase; **gluconeogenesis.**
	**GLYCOLYSIS**		
**0.2**	gb|AAA80675.1| fructokinase [Beta vulgaris]	**1.8**	emb|CAA53507.1| phosphoglucomutase [*Spinacia oleracea*]
		**2.4**	sp|P22418.2|F16P1_SPIOL RecName: Full=Fructose-1,6-bisphosphatase
		**4.4**	ref|XP_001351443.1| phosphoglycerate mutase
		**5.4**	glyceraldehyde-3-phosphate dehydrogenase; glycolysis
		**2.3**	gb|AAC18055.1| fructose-6-phosphate 2-kinase /fructose-2,6-bisphosphatase
		**8.5**	sp|P14766.2|F16P2_SPIOL RecName: Full=Fructose-1,6-bisphosphatase
		**4.3**	emb|CAA03982.1| glucose-6-phosphate isomerase [*Spinacia oleracea*]
		**2.5**	gb|AAB61592.1| fructose-biphosphate aldolase [*Mesembryanthemum* spp.]
		**3.1**	gb|AAR24912.1| fructokinase 3 [*Solanum lycopersicum*]

		**4.9**	sp|P29356.1|ALF_SPIOL RecName: Full=Fructose-bisphosphate aldolase
		**9.9**	sp|P29409.2|PGKH_SPIOL RecName: Full=Phosphoglycerate kinase
	**SUGAR TRANSPORT**		
		**1.5**	gb|AAB68029.1| **putative sugar transporter; member of major facilitator family**
		**1.6**	N3 - **Bidirectional sugar transporter SWEET** – [*Glycine max*]
		**22.6**	emb|CAA58730.1| **sucrose/proton-symporter** [*Beta vulgaris*]
		**3.4**	emb|CAB52689.1| **hexose transporter** [*Solanum lycopersicum*]
		**17.1**	gb|AAF86907.1|AF223359_1 p**hosphoenolpyruvate/phosphate translocator**
	**DEFENSE**		
**0.4**	gb|AAY15221.1| antifungal protein [*Sinapis* alba]	**1.6**	gb|ABK58702.1| **anti-microbial protein 2 [*Amaranthus caudatus*]**
**0.1**	ref|NP_192169.1| ATMLO1/MLO1 (MILDEW RESISTANCE LOCUS O 1)	**7.8**	gb|AAW48295.1| **pore-forming toxin-like protein Hfr-2** [*Triticum aestivum*]
**0.4**	sp|P82010.1|AX2_BETVU RecName: Full=Antifungal protein	**1.8**	emb|CAB56619.1| phragmoplastin [*Nicotiana tabacum*]; **callose deposition**
**0.5**	gb|AAP03878.1| Avr9/Cf-9 rapidly elicited protein 189 [*Nicotiana* spp.]	**2.5**	gb|AAR26482.1| **harpin binding protein 1** [*Malus x domestica*]
**0.3**	dbj|BAB86893.1| syringolide-induced protein 1-3-1B [*Glycine max*]	**3.2**	gb|ACK38253.1| **pathogenesis-related protein** [*Tamarix hispida*]
**0.1**	gb|AAB48305.1| Hs1pro-1 [*Beta procumbens*]	**1.5**	gb|AAC61805.1| **Pto kinase interactor 1** [*Solanum lycopersicum*]
**0.3**	gb|AAD13216.1| latex-abundant protein [*Hevea brasiliensis*]	**2.6**	ref|NP_973870.1| **pathogenesis-related thaumatin family protein**
**0.3**	gb|ABS01349.1| hypersensitive-induced response protein [*Carica papaya*]	**21.3**	dbj|BAA22966.1| **chitinase [*Chenopodium amaranticolor*]**
**0.5**	emb|CAJ44458.1| ALY protein [*Nicotiana benthamiana*] 197 3e-48	**5.5**	sp|P42820.1|CHIP_BETVU RecName: Full=Acidic endochitinase SP2
	**AMINES/ AMINOACID METABOLISM/ SYNTHESIS**		
**0.1**	gb|ABN08767.1| Amine oxidase [*Medicago truncatula*] 594 e-168	**3.1**	emb|CAA45022.1| aspartate aminotransferase [*Panicum miliaceum*]
**0.5**	gb|ABI35986.1| methionine synthase [*Phelipanche ramosa*]	**1.8**	gb|AAA18948.1| ferredoxin-dependent **glutamate synthase** (**GLUTAMATE**)
		**1.9**	gb|AAC19395.1| cystathionine gamma-synthase [*Mesembryanthemum* spp.] (METHIONINE)
		**1.8**	gb|ABW76425.1| **branched-chain aminotransferase** [*Nicotiana benthamiana*]
		**4.3**	gb|ABS72164.1| acetolactate synthase [*Amaranthus hypochondriacus*]; **branched amino acids**
		**5.3**	gb|AAD31898.1|AF145480_1 glutamine synthetase leaf
		**2.2**	gb|AAK14401.1|AF343667_1 cytosolic glutamine synthetase [*Beta vulgaris*]
		**6.1**	sp|Q00834.1|CYSK_SPIOL RecName: Full=Cysteine synthase.
		**5.7**	emb|CAA47329.1| cysteine synthase [*Spinacia oleracea*]
		**18.8**	sp|P93263.1|METE_MESCR RecName: 5-methyltetrahydropteroyltriglutamate--homocysteine methyltransferase; methionine synthesis

	**ANTIOXIDANTS**		
**0.1**	gb|ABO77632.1| **peroxidase** [*Medicago truncatula*]	**3.6**	gb|ABA00455.1| **MnSOD** [*Gossypium hirsutum*]
**0.2**	gb|AAF63025.1|AF244922_**1 peroxidase prx13** precursor [*Spinacia oleraceae*]	**7.3**	gb|ABL60875.1| **germin-like protein 6** [*Vitis vinifera*]
**0.3**	emb|CAA71494.1| **peroxidase** [*Spinacia oleracea*]	**2.3**	dbj|BAF36564.1| **alternative oxidase** [*Nicotiana tabacum*]
**0.5**	emb|CAA71495.1| **peroxidase** [*Spinacia oleracea*]	**3.1**	ref|NP_181046.1| ATO1 (**Arabidopsis THIOREDOXIN O1**)
		**3.1**	dbj|BAA08535.1| **ascorbate peroxidase** [*Spinacia oleracea*]
		**4.5**	dbj|BAG84436.1| NADP-dependent isocitrate dehydrogenase
		**1.9**	emb|CAC82727.1| **monodehydroascorbate reductase** [*Mesembryanthemum* spp.]
		**4.2**	ref|NP_567725.1| ACD1-LIKE; electron carrier [Arabidopsis thaliana]
		**2.8**	ref|NP_568630.1| ATARD4; acireductone dioxygenase
		**4.6**	ref|NP_849444.1| ACD1-LIKE; electron carrier [*Arabidopsis thaliana*]
		**1.9**	sp|Q04522.3|GSTF_SILCU RecName: Full=**Glutathione S-transferase**
		**3.1**	emb|CAA57973.1| class III ADH, **glutathione-dependent formaldehyde dehydrogenase**
		**4.6**	emb|CAK22416.1| **cationic peroxidase** [*Beta vulgaris*]
		**2.4**	emb|CAA71491.1| peroxidase [*Spinacia oleracea*]
		**12.7**	gb|AAK67359.2|AF390210_1 **catalase** [*Suaeda maritima* subsp. *salsa*)
		**8.9**	gb|AAO61856.1| **glutathione S-transferase Z1** [*Malva pusilla*]
		**6.3**	ref|NP_180873.1| KAT2/PED1 (PEROXISOME DEFECTIVE 1)
		**5.8**	sp|O23814.1|GPX4_SPIOL RecName: Full= **Probable phospholipid hydroperoxide glutathione peroxidase**
		**10.8**	emb|CAC17803.1| **peroxiredoxin** [*Phaseolus vulgaris*]
		**6.3**	gb|AAA99518.1| **ascorbate peroxidase**
		**6.4**	emb|CAA71490.1| **peroxidase** [*Spinacia oleracea*]
		**7.4**	sp|P22233.1|SODC_SPIOL RecName: Full=**Superoxide dismutase [Cu-Zn]**
		**13**	gb|AAS92464.1| **Fe-superoxide dismutase 1 precursor** [*Lotus japonicus*]
		**18.4**	emb|CAE22480.1| **superoxide dismutase [Fe] [Solanum** [*Solanum lycopersicum*]
		**53.8**	gb|AAC19398.1| **root catalase** [*Mesembryanthemum crystallinum*]
	**PROTEIN DEGRADATION/ MODIFICATION**		
**0.3**	tpe|CAG29028.1| TPA: putative cystatin [*Zea mays*]	**11**	gb|ABQ10199.1| **cysteine protease** Cp1 [*Actinidia deliciosa*]
		**9.7**	gb|ABQ10204.1| **cysteine protease Cp6** [*Actinidia deliciosa*]
		**33.7**	dbj|BAD29954.1| **cysteine protease** [*Daucus carota*]
		**7.9**	emb|CAD59764.1| **cysteine proteinase inhibitor** [*Celosia cristata*]
		**9.0**	gb|AAF61440.1|AF138264_1 papain-like cysteine proteinase isoform.
		**2.2**	gb|ABD28731.1| C2; **Peptidase, cysteine peptidase** active site

**0.1**	dbj|BAC66627.1| F-box [*Prunus mume*]	**1.6**	sp|O24361.1|PSB5_SPIOL RecName: Full=**Proteasome subunit beta**
**0.2**	gb|ACL14907.1| L-isoaspartyl methyltransferase 2 TIS II alpha-om.	**2.1**	ref|NP_568892.1| **FTSH9 (FtsH protease 9)**; ATP-dependent peptidasase
**0.1**	pir||JC5562 trypsin inhibitor ETIb precursor - *Erythrina variegata*	**20.1**	dbj|BAB20972.1| **aspartic proteinase 4** [*Nepenthes alata*]
**0.5**	sp|O49939.1|TLP40_SPIOL RecName: Full=Peptidyl-prolyl cis-trans isomerase, chloroplastic PPIases accelerate the folding of proteins**. It catalyzes the cis-trans isomerization of proline imidic peptide bonds in oligopeptides.** Has a regulatory effect on thylakoid protein phosphorylation.		
**0.5**	gb|ABI84264.1| **ubiquitin-conjugating enzyme** [*Arachis hypogaea*]		
**0.1**	emb|CAC86004.1| **aspartic proteinase** [*Theobroma cacao*]		
**RNA/ DNA DEGRADATION/ MODIFICATION/ EPIGENETIC CONTROL**			
**0.2**	ref|NP_195467.1| ribonuclease III family protein [*Arabidopsis thaliana*]	**4.2**	gb|AAM62421.1|AF515795_1 Drm3 [*Pisum sativum*; Encodes DRM3 (Domains Rearranged Methyltransferase3), a catalytically mutated paralog of the cytosine methyltransferase DRM2. Despite being catalytically mutated, DRM3 is required for normal maintenance of non-CG DNA methylation: **Chromatin-Based Epigenetic Regulation of Plant Abiotic Stress Response.**
**0.5**	ref|NP_565843.1| inosine-uridine preferring nucleoside hydrolase	**1.6**	emb|CAC43291.1| putative linker histone H1 variant protein [*Beta vulgaris*]
**0.3**	ref|XP_002171493.1| DNA ligase [*Schizosaccharomyces japonicas*]	**2.1**	gb|ACK75561.1| allantoate amidohydrolase [*Glycine max*]; **recycling purine-ring nitrogen in all plants**
**0.4**	gb|ACG39790.1| pre-mRNA-splicing factor 19 [*Zea mays*]	**1.7**	gb|ABD32395.1| **Histone-fold/TFIID-TAF/NF-Y** [*Medicago truncatula*]
**0.1**	gb|AAP31307.1| histone H1 [*Lens nigricans*]	**2.9**	sp|P0C2M6.1|DED1_EMENI RecName: Full=ATP-dependent RNA helicase.
		**7.1**	ref|NP_001078516.1| **histone H3.2** [*Arabidopsis thaliana*]
**LIPID MODIFICATION/ DEGRADATION**			
**FATTY ACID DESATURASES**			
		**6.9**	gb|AAM63459.1| **putative delta 9 desaturase** [*Arabidopsis thaliana*]
		**3.6**	gb|ABG88131.1| **chloroplast omega-3 fatty acid desaturase**
		**3.6**	gb|ABL61262.1| **chloroplast omega-3 desaturase** [*Portulaca oleracea*]
		**4.2**	emb|CAK54360.1| putative desaturase-like protein [*Trifolium repens*]
		**8.7**	gb|ABS86965.1| **delta-9 fatty acid desaturase** [*Descurainia sophia*]
**WAX SYNTHESIS**			
		**2.7**	gb|AAZ08051.1| **wax synthase** [*Petunia x hybrida*]
		**1.5**	gb|AAY23355.1| 3-ketoacyl-CoA reductase 2 [*Gossypium hirsutum*] ; **Very-long-chain fatty acids (VLCFAs) are essential precursors of cuticular waxes and aliphatic suberins in roots.**

**0.1**	gb|ABD32376.1| Lipolytic enzyme, G-D-S-L [*Medicago truncatula*]	**3.7**	sp|P52877.1|SERC_SPIOL RecName: Full=Phosphoserine aminotransferase
**0.1**	gb|AAP55714.1| **GDSL-lipase** [*Chenopodium rubrum*]	**2.1**	gb|AAB53764.1| aminoalcohol phosphotransferase [*Brassica rapa*], **phospholipids**
**0.5**	ref|ZP_01692219.1| glycerophosphoryl diester phosphodiesterase		
	**SIGNALING**		
	**KINASES/ PHOSPHATASES/ OTHERS**		
**0.1**	emb|CAB82852.1| **protein kinase MK6** [*Mesembryanthemum crystallinum*]	**4.6**	ref|NP_199595.1| AMK2 (ADENOSINE MONOPHOSPHATE KINASE); **AMPK plays critical roles in regulating growth and reprogramming metabolism.**
**0.1**	emb|CAB89490.1| CRK1 protein [*Beta vulgaris* subsp. *vulgaris*]; The CDPK-related kinases (CRKs) are a type of serine-threonine kinases belonging to the CDPK-SnRK superfamily.		
**0.1**	gb|AAK62444.1|AF386999_1 similar to wpk4 protein kinase [*Arabidopsis thaliana*]; The first identified serine/threonine protein kinase, which according to Halford and Hardie (1998) **belongs to the SnRK3 subfamily, was WPK4 from wheat.**		
**0.1**	emb|CAC82999.1| **calcium-dependent protein kinase 3** [*Nicotiana tabacum*]		
**0.2**	emb|CAC24490.1| choline kinase [*Pisum sativum*]		
**0.4**	gb|AAD31900.1|AF145482_1 putative serine/threonine protein kinase		
**0.3**	gb|ABW80999.1| PI-phospholipase C PLC5 [Solanum lycopersicum]	**1.7**	emb|CAC10358.1| **protein phosphatase 2C** [*Nicotiana tabacum*]
		**3.4**	gb|AAC36700.1| **protein phosphatase-2C**; PP2C [*Mesembryanthemum* spp.]
		**2.3**	gb|AAD11430.1| **protein phosphatase 2C homo** [*Mesembryanthemum* spp.]
**0.3**	ref|NP_201227.3| calmodulin-binding protein [*Arabidopsis thaliana*]	**2.1**	sp|O81919.1|CALR_BETVU RecName: Full=Calreticulin;
**0.2**	gb|ABM55247.1| calmodulin-binding protein [*Beta vulgaris*]		
	**RECEPTOR-LIKE KINASES**		
**0.2**	gb|AAL40864.1| receptor protein kinase-like protein [*Capsicum annuum*]		
**0.2**	gb|ABY40731.1| FERONIA receptor-like kinase [*Poncirus trifoliata*]		
**0.2**	dbj|BAE46451.1| putative receptor protein kinase PERK1 [Glycine]; **The proline-rich, extensin-like receptor kinase-1; wound-inducible.**		
**0.1**	ref|NP_196408.2| leucine-rich repeat family protein	**5.7**	ref|NP_175397.1| leucine-rich repeat family protein [*Arabidopsis thaliana*]
	**INOSITOL METABOLISM**		
**0.4**	sp|O49071.1|IMPP_MESCR RecName: Full=**Inositol monophosphatase**		
**0.5**	gb|AAL28131.1|AF433879_1 **myo-inositol-1-phosphate synthase** [*Suaeda salsa*]		
	**PHOTOSYNTHESIS/ ENERGY/ PHOTORESPIRATION**		
**0.3**	sp|Q9XGX4.1|RBS3_AMAHP RecName: Full=**Ribulose bisphosphate carboylase**	**1.7**	sp|P16016.2|CAHC_SPIOL RecName: Full=Carbonic anhydrase, chloroplast

**0.3**	emb|CAK18840.1| **subunit IV of photosystem I** (PSI-E) precursor	**6.6**	sp|P37224.1|MAOM_AMAHP RecName: Full=NAD-dependent malic enzyme
**0.4**	emb|CAA04157.1| **Rieske iron-sulfur protein Tic55** [*Pisum sativum*]	**5.4**	sp|P52765.1|NU3C_LUPLU RecName: Full=**NAD(P)H-quinone oxidoreductase**
**0.3**	ref|NP_194457.2| **quinone reductase** family protein [Arabidopsis t.]	**2.5**	sp|P00289.2|PLAS_SPIOL RecName: Full=Plastocyanin, chloroplastic
**0.3**	sp|Q41014.2|FENR2_PEA RecName: Full=**Ferredoxin--NADP reductase**	**1.6**	gb|AAL77589.1|L43068_1 chloroplast ribose-5-phosphate isomerase
		**3.0**	emb|CAA29590.1| **Rieske FeS-precursor** [*Spinacia oleracea*]
		**3.2**	sp|P10690.1|PSBR_SPIOL RecName: Full=**Photosystem II 10 kDa polypeptide**
		**1.7**	gb|AAT02656.1| mitochondrial pyruvate dehydrogenase kinase isoform
		**2.0**	sp|P41348.2|FTRC1_SPIOL RecName: Full=**Ferredoxin-thioredoxin reductase**
		**15.1**	gb|AAM78591.1| **Rubisco activase** [*Chenopodium quinoa*]
		**1.9**	sp|Q9SM43.2|VDE_SPIOL RecName: Full=**Violaxanthin de-epoxidase**
		**2.4**	sp|P12357.1|PSAG_SPIOL RecName: Full=**Photosystem I reaction center**
		**1.7**	ref|NP_189086.1| HCF101 (HIGH-CHLOROPHYLL-FLUORESCENCE 101)
		**2.1**	gb|ABD52327.1| **plastid protoporphyrinogen oxidase** [*Amaranthus* ssp.]
		**3.2**	gb|AAB40396.1| **glycolate oxidase** [*Mesembryanthemum crystallinum*]
		**3.1**	gb|ACK56136.1| transaldolase [*Dimocarpus longan*]; Calvin cycle
		**7.5**	gb|AAB95218.1| putative **serine-glyoxylate aminotransferase**
		**2.4**	gb|ABA54865.1| putative 3-**deoxy-D-arabino-heptulosonate 7-phosphate phosphatase**
		**3.6**	gb|ACF06473.1| light-inducible protein ATLS1 [*Elaeis guineensis*]
		**5.0**	emb|CAA04526.1| **magnesium chelatase subunit** [*Glycine max*]
		**2.7**	emb|CAC43293.1| putative **phosphoenolpyruvate carboxylase kinase**
		**2.1**	ref|NP_191434.1| CSY2 (CITRATE SYNTHASE 2)
		**2.4**	gb|ACG31979.1| **mo-molybdopterin cofactor sulfurase** [*Zea mays*]
		**3.1**	ref|NP_181092.1| plastid-lipid associated protein PAP
		**9.9**	sp|P05435.2|ATPG_SPIOL RecName: Full=**ATP synthase gamma chain**
		**5.9**	ref|NP_564093.1| wound-responsive family protein [*Arabidopsis thaliana*] sp|O20250.1|TKTC_SPIOL RecName: Full=**Transketolase, chloroplastic**; Calvin cycle
		**3.1**	sp|P21239.2|RUB1_BRANA RecName: Full=**RuBisCO large subunit**
		**2.6**	emb|CAJ77390.1| **major chlorophyll a/b binding protein LHCb1.2**
		**8.0**	sp|P12355.1|PSAF_SPIOL RecName: Full=**Photosystem I reaction center subunit III, chloroplastic.**
		**4.6**	emb|CAA96568.1| **CP12** [*Spinacia oleracea*]

		**6.5**	ref|NP_054960.1| **photosystem II 47** kDa protein [*Spinacia olerace*]
		**8.1**	sp|P31853.2|ATPX_SPIOL RecName: Full=**ATP synthase B' chain**, chloroplastic
		**12.5**	gb|AAC24709.1| **ribulose-phosphate 3-epimerase transit.**
		**3.9**	dbj|BAE07183.1| putative **serine decarboxylase** [*Beta vulgaris*]
		**12.8**	sp|P50433.1|GLYM_SOLTU RecName: Full=**Serine hydroxymethyltransferase**
		**17**	sp|P12301.1|PSBQ_SPIOL RecName: Full=**Oxygen-evolving enhancer protein**
		**13.7**	sp|Q41385.1|PSAL_SPIOL RecName: Full= **Photosystem I reaction center subunit XI, chloroplastic**
		**27.9**	sp|Q42910.1|PPDK_MESCR RecName: Full=**Pyruvate, phosphate dikinase**
		**6.5**	gb|ABO61376.1| **serine hydroxymethyltransferase** [*Populus tremuloides*]
		**14.1**	ref|YP_319761.1| **photosystem II 44 kDa** protein [*Acorus calamus*]
		**21**	ref|NP_064080.2| NADH dehydrogenase subunit 4 [*Beta vulgaris*]
**TRANSCRIPTION/ TRANSLATION**			
		**2.2**	gb|AAT01416.1| translation initiation factor 5A [*Tamarix* sp.]
		**3.3**	sp|Q43467.1|EFTU1_SOYBN RecName: Full=Elongation factor Tu, chloroplast
		**3.4**	ref|NP_173165.1| FUG1 (FU-GAERI1); translation initiation factor
		**3.8**	sp|O23755.1|EF2_BETVU RecName: Full=Elongation factor 2.
		**7.5**	gb|ACF06581.1| translational initiation factor eIF1 [Elaeis guinea]
**0.1**	gb|ABO31952.1| ribosomal protein S3 [*Peridiscus lucidus*]	**2.3**	sp|O80360.1|RK3_TOBAC RecName: Full=50S ribosomal protein L3,
**0.3**	gb|AAT39969.1| 60S ribosomal protein L34, putative [*Solanum demisum*]	**2.9**	gb|AAA34039.1| ribosomal protein 30S subunit
**0.4**	gb|ACF06518.1| 40S ribosomal protein S23 [*Elaeis guineensis*]	**2.1**	gb|AAL91663.1| 60s acidic ribosomal protein [*Prunus dulcis*]
**0.4**	sp|Q9ST69.1|RR5_SPIOL RecName: Full=30S ribosomal protein S5	**3.5**	emb|CAA63651.1| ribosomal protein L4 [*Spinacia oleracea*]
**0.5**	gb|AAY41426.1| putative L24 ribosomal protein [*Ipomoea batatas*]	**2.7**	sp|P29344.1|RR1_SPIOL RecName: Full=30S ribosomal protein S1, chloroplast
		**3.4**	emb|CAG47084.1| 40S ribosomal protein S9 [*Catharanthus roseus*]
		**4.9**	ref|NP_563847.1| 60S ribosomal protein L21 (RPL21A) [*Arabidopsis thaliana*]
		**4.9**	ref|NP_187574.1| 60S ribosomal protein L4/L1 (RPL4A) [*Arabidopsis thaliana*]
**0.1**	ref|NP_001099678.1| poly(A) binding protein, cytoplasmic 3		
**OTHERS**			

**0.1**	gb|ABD65058.1| GRF zinc finger containing protein [*Brassica oleraceae*]	**3.3**	gb|AAC18788.1|AAC18788 Contains similarity to beta scruin gb
**0.3**	gb|ABC88375.1| Smad4 [*Nematostella vectensis*] 35 3.0	**4.9**	ref|NP_192259.1| ankyrin repeat family protein [*Arabidopsis thaliana*]
**0.1**	ref|NP_568721.1| short-chain dehydrogenase/reductase (SDR) family	**2.7**	gb|AAL57201.1|AF355597_1 putative nodule membrane protein [*Medicago* spp.]
**0.4**	gb|AAO43000.1| early tobacco anther 1 [*Nicotiana tabacum*]	**2.6**	gb|AAZ85265.1| CT099 [*Lycopersicon pimpinellifolium*]
**0.1**	gb|ABD98040.1| epicotyl-specific tissue protein [*Striga asiatica*]		
**0.1**	emb|CAA66109.3| specific tissue protein 2 [*Cicer arietinum*]		
**0.1**	gb|ACG30449.1| PGPS/D12 [Zea mays]		
**0.1**	ref|NP_973902.1| NHL repeat-containing protein [*Arabidopsis thaliana*]		
**0.2**	gb|ABL86674.1| PDF [*Gossypium barbadense*]		
**0.5**	gb|AAY51975.1| GIR1 [*Vigna radiata*]		
**0.5**	ref|NP_190741.1| saposin B domain-containing protein [*Arabidopsis thaliana*]		
**0.5**	gb|ACG42167.1| DAG protein [*Zea mays*]		
**0.1**	gb|AAK71662.1|AF395901_1 mature anther-specific protein LAT61		
**0.2**	gb|ACJ02356.1| GAST-like protein [*Populus euphratica*]		
**0.3**	ref|NP_680660.2| BTB/POZ domain-containing protein [*Arabidopsis thaliana*]		
	gb|ABD83324.1| Fgenesh protein 108 [*Beta vulgaris*] 49 0.001		
**0.5**	gb|AAD22104.1| B12D protein [*Ipomoea batatas*]		
**0.4**	ref|NP_564385.1| NFD3 (NUCLEAR FUSION DEFECTIVE 3)		
